# Non-Invasive Low Pulsed Electrical Fields for Inducing BBB Disruption in Mice—Feasibility Demonstration

**DOI:** 10.3390/pharmaceutics13020169

**Published:** 2021-01-27

**Authors:** Shirley Sharabi, David Last, Dianne Daniels, Ido Didi Fabian, Dana Atrakchi, Yael Bresler, Sigal Liraz-Zaltsman, Itzik Cooper, Yael Mardor

**Affiliations:** 1 The Advanced Technology Center, Sheba Medical Center, Tel Hashomer, Ramat-Gan 5262000, Israel; david.last@sheba.health.gov.il (D.L.); Dianne.daniels@sheba.health.gov.il (D.D.); yael.bresler@sheba.health.gov.il (Y.B.); yael.mardor@sheba.health.gov.il (Y.M.); 2 Goldschleger Eye Institute, Sheba Medical Center, Tel Hashomer, Ramat-Gan 5262000, Israel; didi@didifabian.com; 3 Sackler School of Medicine, Tel-Aviv University, Tel-Aviv 69978, Israel; 4 The Joseph Sagol Neuroscience Center, Sheba Medical Center, Tel Hashomer, Ramat Gan 52621, Israel; Dana.Atrakchi@sheba.health.gov.il (D.A.); Sigal.LirazZaltsman@sheba.health.gov.il (S.L.-Z.); itzik.cooper@sheba.health.gov.il (I.C.); 5 Department of Pharmacology, Institute for Drug Research, Hebrew University, Jerusalem 9112001, Israel; 6 Institute for Health and Medical Professions, Department of Sports Therapy, Ono Academic College, Kiryat Ono 5545173, Israel; 7 Interdisciplinary Center Herzliya, Herzliya 4610101, Israel

**Keywords:** blood–brain barrier disruption, pulsed electrical fields, MRI, treatment response assessment maps, non-invasive, neurodegenerative diseases

## Abstract

The blood–brain barrier (BBB) is a major hurdle for the treatment of central nervous system disorders, limiting passage of both small and large therapeutic agents from the blood stream into the brain. Thus, means for inducing BBB disruption (BBBd) are urgently needed. Here, we studied the application of low pulsed electrical fields (PEFs) for inducing BBBd in mice. Mice were treated by low PEFs using electrodes pressed against both sides of the skull (100–400 square 50 µs pulses at 4 Hz with different voltages). BBBd as a function of treatment parameters was evaluated using MRI-based treatment response assessment maps (TRAMs) and Evans blue extravasation. A 3D numerical model of the mouse brain and electrodes was constructed using finite element software, simulating the electric fields distribution in the brain and ensuring no significant temperature elevation. BBBd was demonstrated immediately after treatment and significant linear regressions were found between treatment parameters and the extent of BBBd. The maximal induced electric field in the mice brains, calculated by the numerical model, ranged between 62.4 and 187.2 V/cm for the minimal and maximal applied voltages. These results demonstrate the feasibility of inducing significant BBBd using non-invasive low PEFs, well below the threshold for electroporation.

## 1. Introduction

Despite the rise in the prevalence of neurodegenerative diseases in recent years, mainly due to the increasing lifespan, drug development for central nervous system (CNS) disorders such as stroke, brain tumors and neurodegenerative disorders such as Alzheimer’s disease, amyotrophic lateral sclerosis and Parkinson’s disease is extremely challenging. The reasons include brain complexity, side effects and the blood–brain barrier (BBB) which prevents most therapeutics from reaching the CNS [1].

The BBB is a multicellular barrier composed of brain endothelial cells as well as pericytes and astrocytes. The brain microvasculature endothelial cells have highly developed tight junctions and adherent junctions complexes which together with a wide range of efflux pumps limit transport of up to 98% of small molecules and almost 100% of large molecules form the blood to the brain tissue [2]. Although essential for normal brain function, the BBB is a significant obstacle for the treatment of brain diseases as it limits the passage of most therapeutic agents from the blood stream into brain tissue.

Research for CNS drug delivery focuses on three main approaches [3]: (1) reformulating existing drugs to cross the BBB by physiological transport mechanisms and drug-delivery approaches such as microspheres and colloidal drug-carriers [4]; (2) minimally invasive/invasive local delivery methods such as convection-enhanced delivery [5,6] and biodegradable wafers; and (3) disrupting the BBB, where non-localized methods include osmotic BBB disruption (BBBd) [7], while localized approaches include laser interstitial thermotherapy, MRI-guided focused ultrasound (MRgFUS) [8] and electroporation (EP) [9].

During EP, high pulsed electrical fields (PEFs), in the order of hundreds to thousands volts per centimeter (V/cm), are applied using at least two electrodes, destabilizing the cell membrane and inducing nano-scale pores in the cell’s membranes. When the electric fields are applied such that the pores can reseal within few minutes, the treatment is termed reversible EP and is used to increase the uptake of therapeutic molecules. When cell membranes are permeabilized in a manner leading to cellular death [10], it is called irreversible EP. Both reversible and irreversible EP are methods in different stages of development for treating tumors outside and inside the CNS [11,12,13,14,15,16].

It has been recently demonstrated that EP can induce transient BBBd in vivo, thus enhancing intraparenchymal drug uptake. Such treatments showed improved survival and reduced tumor volume or growth rates in glioma-bearing rats and dogs [17,18,19,20]. The electric field threshold for inducing BBBd by EP was shown to be 500–700 V/cm depending on treatment parameters [17,19]. Recently, Lorenzo et al. [21] demonstrated that high-frequency EP (H-FRE, delivery of high numbers of short bipolar pulses at 250–500 kHz) also induces BBBd. The treatment was conducted using two needle electrodes inserted into the brain parenchyma with a voltage to distance ratio of 600 V/cm. The authors determined the threshold for BBBd to be 113 V/cm. These treatments, although showing minimal side effects, require at least one intracranial electrode and often require craniotomy. Moreover, when EP is used for inducing large BBBd volumes, irreversible EP may occur, resulting in brain damage [17,18,22].

We recently introduced the application of low PEFs, in the order of 15–100 V/cm, for inducing BBBd in vitro well below the threshold for EP [23]. Unlike EP, which induces transcellular BBBd, this method was found to induce paracellular BBBd.

In the current study, we aimed to demonstrate the feasibility of applying non-invasive (intact skull) low PEFs for inducing BBBd in naïve mice. A quantification method using MRI-based treatment response assessment maps (TRAMs) [24,25] was developed. The TRAMs were applied in order to quantify the effects of treatment protocols on the volume and intensity of BBBd [20].

## 2. Methods

### 2.1. Animals

The study was approved by the Animal Care and Use Committee of Sheba Medical Center and was performed in accordance with the guidelines of ARRIVE.

Sixty-four Hsd mice, weighting 25–30 g, were used in this study. A 12:12-h light–dark cycle was maintained. Food and water were provided ad libitum. Fifty-eight mice underwent PEFs treatment followed by delayed contrast MRI and euthanasia or Evans blue (EB) infusion followed by delayed perfusion and brain extraction, for BBBd assessments. An additional six mice underwent PEFs treatment and were kept for observation. The mice were scanned by MRI 48 h post-treatment for assessment of treatment-related toxicity.

### 2.2. MRI Experimental Outline

Anesthesia was administered by intramuscular injections of 250 µL of 1 mL/kg ketamine and 0.5 mL/kg xylazine. The anesthetized mice were subjected to a midline scalp incision and the skin was separated from both sides to expose the skull. Two stainless steel 1.5 cm square plate electrodes (Caliper Electrode, Harvard Apparatus, Holliston, MA, USA) were pressed against the sides of the intact skull after application of conductive gel. The distance between the electrodes was 1.2–1.3 cm.

The mice were treated by 100–400 square pulses with a pulse duration of 50 µs at 100–300 V. The pulses were applied in sets of 25 pulses at a frequency of 4 Hz with 5-s intervals between sets. Sham procedures for the control rats included anesthesia, skin incision, placing the electrodes and leaving the electrodes in place for 60 s. At 2–5 min post-PEFs application, the mice were scanned by MRI to evaluate BBBd. The MRI contrast agent was injected into the tail vein in the MRI, immediately prior to the first scan.

The MRI experiments were designed to demonstrate the feasibility of inducing BBBd using non-invasive low PEFs and to study the dependency on the treatment parameters. Three to six mice were treated in each treatment group (100 V with 100, 200, 300 and 400 pulses; 150 V with 100, 200, 300 and 400 pulses; 100 pulses with 100, 150, 200 and 300 V), up to a total of 43 mice.

Six mice were divided into two treatment groups of 3 mice each (100 V with 100 pulses and 300 V with 100 pulses) and scanned by MRI 48 h post-treatment to evaluate possible damage.

### 2.3. MRI Data Acquisition

The MRI experiments were performed using a GE Optima MRI (1.5T) with an 8-channel phased array wrist coil. The contrast agent (Gd-DOTA, 0.016 mmol/kg, Dotarem, Guerbert) was injected into the tail vein immediately prior to the scan. The mice were scanned with the following MR sequences: repeated 3D contrast-enhanced T1-weighted MRI (T1-MRI) for at least 30 min after contrast injection to depict BBBd, T2-weighted MRI (T2-MRI) to depict edema or damage and T2*-weighted gradient echo (GE) and susceptibility-weighted MRI (SWAN) to depict potential bleeding. MRI sequences were acquired with the following parameters: 3D Cube T1-MRI: 10 cm FOV (phase FOV: 0.8), 244.14 kHz bandwidth, TE/TR = 21.4/602 ms, 0.8 mm slice thickness, 2562 matrix size, resulting in a voxel size of 0.39 × 0.39 × 0.4 mm^3^ after zerofill; 2D fast spin echo T2-MRI: 12 cm FOV (phase FOV: 0.5), 1.0 mm slice thickness, 20.83 kHz bandwidth, TE/TR = 85/5300 ms, 256 × 224 matrix size, resulting in a voxel size of 0.43 mm^3^ after zerofill. GE images were acquired with a 256 × 224 matrix, 12 cm FOV, TE/TR = 15/300 ms, BW = 15.63 kHz, NEX = 2, a flip angle of 15° and 1 mm slice thickness. The in-plane voxel size was (0.47 mm^2^). 3D SWAN: 10 cm FOV (phase FOV: 0.8), 1.0 mm slice thickness, 31.25 kHz bandwidth, TE/TR = 47/124 ms, 30 degrees flip angle, 320 × 256 matrix size, resulting in a voxel size of 0.43 mm^3^ after zerofill.

### 2.4. Image Analysis

**Figure 1:**
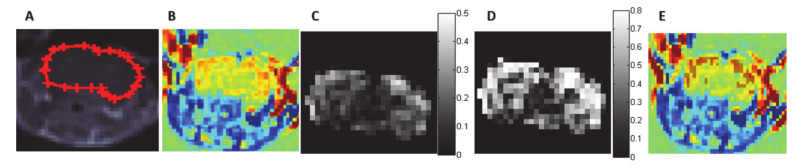
Examples of calculated maps: (**A**) A T1-MRI slice with the plotted brain region of interest (ROI) overlaid. (**B**) Treatment response assessment maps (TRAMs) calculated by subtracting the 1st series post-contrast from the series obtained 30 min post-contrast injection showing red (contrast accumulation) regions (demonstrating higher blood–brain barrier disruption (BBBd) in the cortex). (**C**) Maximal intensity increase map in the same brain slice. (**D**) r^2^ map depicting the fit r^2^ of each pixel in the same brain slice. (**E**) Pixels included in the BBBd ROI (intensity increase > 5% and r^2^ > 0.6), marked in brown and overlaid on the TRAMs.

For each mouse, TRAMs were calculated for visualization of subtle BBBd using Matlab (R2014a, Mathworks, Natick, MA, USA) as previously described [20]. In short, all T1-MRI series were registered to the 1st post-contrast T1-MRI series using 3D rigid registration. The 1st series post-contrast was than subtracted from each of the delayed series generating TRAMs. In the TRAMs, contrast clearance, resulting in a negative signal, was depicted blue and contrast accumulation, resulting in a positive signal, was depicted red. No change was depicted green (Figure 1B).

For quantification of BBBd volumes and intensities, a pixel by pixel analysis was conducted. First, a region of interest (ROI) was plotted over all the brain slices so that the entire brain was segmented for each mouse (Figure 1A) for all time points post-contrast injection. Next, within the brain region, the intensity of each pixel was plotted as a function of time post-contrast injection and fitted to a 2-exponential function, based on a two-compartment exchange model of Tofts et al. [26,27,28]. The fit resulted in quantification of the following parameters: time to peak, maximal intensity increase, wash-in and wash-out slopes and coefficient of determination (r^2^). Then, maps of the maximal intensity increase and r^2^ were generated (Figure 1C,D). Pixels presenting with BBBd were defined as those above a threshold of 1.05 for the intensity increase (representing a >5% increase in signal intensity) and 0.6 for r^2^.

Pixels above these thresholds were included in the BBBd ROI (Figure 1E). Lastly, a cluster analysis was conducted, removing clusters of less than 10 pixels (0.6 mm^3^) from the BBBd ROI in order to minimize inclusion of noise. BBBd volume was then calculated by multiplying the number of pixels in the BBBd ROI by the pixel volume. BBBd intensity was calculated as the mean maximal intensity increase in the BBBd ROI.

### 2.5. Evans Blue Experimental Outline

EB extravasation is the most widely used method to detect BBBd. EB is limited by the BBB; therefore, EB in brain tissue is indicative of BBB permeability changes [29]. The EB experiments were designed to quantify the amount of EB that crosses the BBB in specific brain regions. Fifteen mice were treated with low PEFs in 3 treatment groups of 5 mice each: sham, 100 V with 100 pulses and 200 V with 100 pulses.

The surgical procedure and PEFs treatments were performed similarly to those of the MRI experiments. The experiments were performed using the optimized protocol of Wang et al. [30]. In short, a 2% EB solution (4 mL/kg) was injected into 15 mice tails immediately post-PEFs application and was allowed to circulate for 20 min, after which the mice were thoroughly perfused for 8 min with 60 mL of saline. Following perfusion, brains were dissected (cortex, cerebellum and rest of the brain), weighed in Eppendorf tubes and frozen a −80 °C. Frozen brain samples were thawed and mixed with a 1:3 (W(mg)/V(µL)) ratio of cold 50% trichloroacetic acid (TCA) in 0.9% saline. Samples were homogenized for 5 min (continuous beating) using a pre-cooled metal-bead homogenizer (BULLET BLENDER^®^ BBX24). Tubes were then centrifuged for 20 min at 10,000× *g* at 4 °C. An amount of 30 µL was collected from each sample in duplicates into a clear 96-well plate. An amount of 90 µL of 95% ethanol was then added to each sample and spectroscopically detected by 620/680 nm excitation/emission using a florescence plate reader (Tecan Infinite F200). The amount of EB in each sample was determined from a calibration curve (ranging from 0.083 to 4 µg/mL) prepared with EB that was dissolved in a 50% TCA solution (30 µL) and 90 µL of 95% ethanol and was then normalized to the brain sample weight.

### 2.6. Numerical Modeling

**Figure 2:**
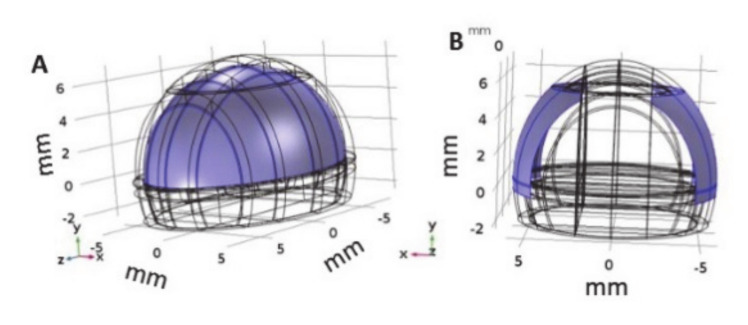
Model geometry and electrodes position (**A**). Geometry of the mice head model with the brain domain in purple. (**B**) Location of the electrodes (purple) where the boundary conditions were applied to.

A 3D finite elements model of a mouse brain and electrodes was constructed using COMSOL (COMSOL Multiphysics 5.3a, Stockholm, Sweden). The mouse head was modeled as an ellipsoid of 6 × 8 × 7 mm semi-axes with the lower seventh removed. The resulting shape had a 12 mm diameter in the coronal plain, a maximal length of 16 mm (sagittal plain) and maximal height of 12 mm (Figure 2A). Additional internal layers were added representing the skull (0.6 mm), dura (0.1 mm) and brain. The final brain volume reached 510.4 mm^3^. These parameters of the head model were obtained by measuring the volume and semi-axes of the head and brain of a mouse scanned by MRI. The number of elements in the finalized model was 666,024 and 211,022 in the brain domain. The tissue electrical and thermal properties were obtained from the literature (Table 1).

The Laplace Equation for electric potential was used to describe the electric field:

(1) $$\nabla \cdot \left(\sigma \nabla \;\varphi \right)=0$$

where *σ* represents the tissue electric conductivity and *φ* is the electric potential. The Dirichlet boundary condition was applied to the surface of the electrodes (Figure 2B) and the Neumann boundary condition was set to zero for the remaining outer surface boundaries as they were considered electrically isolative. The modified Pennes bioheat equation with an additional Joule heating source term was used to determine thermal effects. The outer surface of the model was considered thermally insulted:

(2) $$\nabla \;\cdot \left(k\nabla T\right)\;+\;w_{b}c_{b}\left(T_{a}-T\right)\;+\;Q_{met}+\sigma {\left|\nabla \varphi \right|}^{2}=\rho c_{p}\frac{\partial T}{\partial t}$$

where *k* is the tissue thermal conductivity, *T* is the temperature, w_b_ is the blood perfusion, *c_b_* is the blood heat capacity, *T_a_* is the arterial temperature, *Q_met_* is the metabolic heat generation, *ρ* is the tissue density, *c_p_* is the heat capacity of the tissue, *φ* is the electrical potential and *σ* is the electrical conductivity. The brain temperature prior to treatment was set to 36.8 °C.

The thermal model was solved using a duty cycle approach [36]. In short, instead of calculating the Joule heating for each pulse and implementing the new temperature as the next step initial temperature, a time-dependent solver was applied, and the thermal dissipation was multiplied by the pulse length (50 µs) and the number of pulses. The duty cycle approach does not take into consideration cooling effects during the pulse intervals and thus is considered more conservative in evaluating temperature increase.

### 2.7. Statistical Analysis

Results are presented as means with standard errors. The differences between the sham and the minimal treatment (100 pulses at 100 V) groups were studied by an independent samples t test. The dependency of the extent of BBBd in the number of pulses and treatment voltage was studied using a linear regression analysis [37] both for the MRI experiments and the EB experiments. Linearity, homoscedasticity and normality of the residuals were verified.

## 3. Results

### 3.1. MRI Experiments

At 2–5 min following PEFs treatment, the mice were scanned by MRI. T2-MRI, SWAN and repeated contrast-enhanced 3D T1-MRIs were acquired. The TRAMs were generated for each mouse.

**Figure 3:**
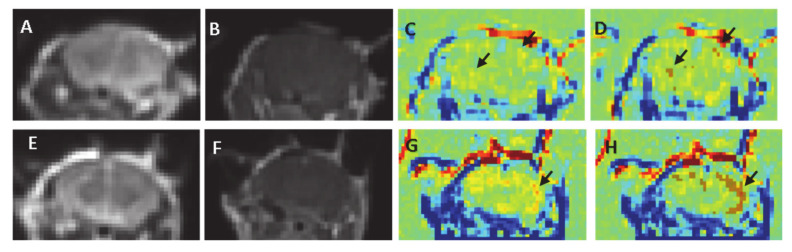
MRIs of a sham mouse and a mouse from the 100 V, 100 pulses group (**A**–**D**). Sham, (**E**,**F**). 100 V, 100 pulses (**A**) + (**D**). Table 2. MRIs showing no signs of damage or edema (**B**) + (**F**). Contrast-enhanced T1-MRI showing no visible enhancement in the brain parenchyma. (**C**) + (**G**). TRAMs calculated by subtracting the first T1-MRI from the 30 min T1-MRI. No clusters of contrast accumulation are visible in the sham mouse ((**C**), arrow shows scattered pixels), while contrast accumulation is visible in the cortex (arrow) of the treated mouse (**G**). (**D**) + (**H**) The results of the analysis algorithm showing random small clusters in the sham brain (brown pixels) and significant BBBd clusters (mainly in the cortex) in the treated mouse.

No signs of edema, damage or bleeding were observed in any of the treated mice. Standard 3D T1-MRI obtained immediately post-contrast injection showed no clear enhancement in the brain parenchyma in any of the mice (Figure 3A,B,E,F). Nevertheless, the calculated TRAMs, which are sensitive to subtle BBBd, reveled significant BBBd which increased with the treatment voltage and with the number of pulses. The TRAMs showed contrast agent accumulation (depicted red) mainly in the cortical region, as predicted by the simulation (Figure 3G–H). The average volume of BBBd found for the sham group (0 V) was 6.08 ± 3.09 mm^3^ and the average intensity increase was 6.76% ± 0.2%. The pixels determined to represent BBBd by our analysis algorithm were sporadic pixels scattered in the brain resulting from noise. The average BBBd volume of the minimal treatment group (100 pulses at 100 V) was significantly larger than that of the sham group (39.10 ± 9.66 mm^3^, *p* < 0.02) and the disrupted volume was concentrated mainly in the cortex of the mice, as can be seen in Figure 3. The increase in signal intensity was also significantly higher than that of the sham group (13.73 ± 4.09, *p* < 0.009). These results suggest that subtle BBBd was obtained when applying 100 pulses at 100 V.

### 3.2. Dependency of BBBd on the Applied Voltage

**Figure 4:**
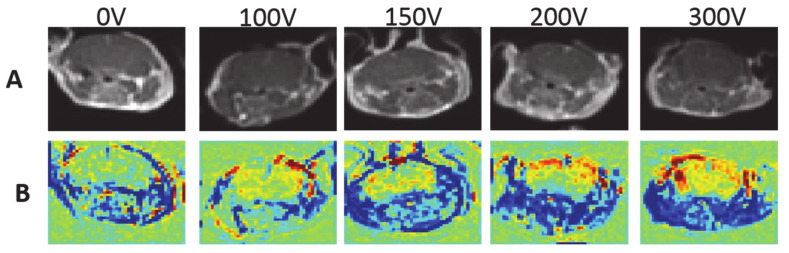
Examples of T1-MRIs and their equivalent 30 min TRAMs for different treatment voltages. (**A**) T1-MRI obtained immediately post-contrast injection for sham (0 V) and for mice treated with 100 pulses at 100–300 V. No enhancement is visible in the brain parenchyma (**B**). TRAMs calculated by subtracting the first T1-MRI post-contrast injection from the 30 min T1-MRI, showing increased BBBd volumes (depicted yellow/red, mainly in the cortex) with increased treatment voltage.

**Figure 5:**
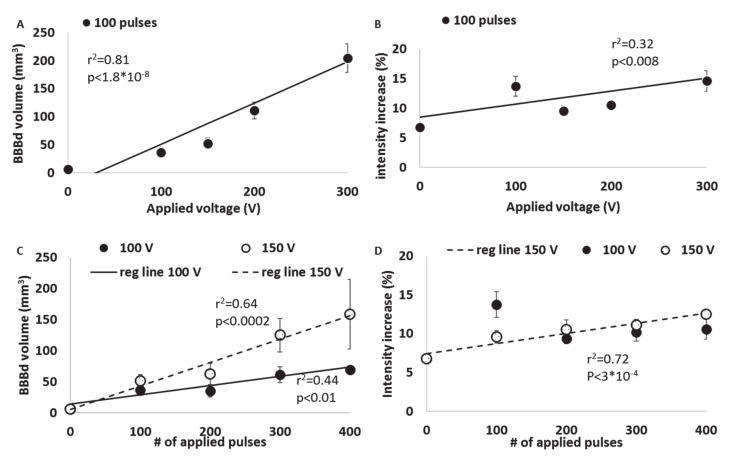
Experimental data and linear regression equation lines describing the dependency of BBBd volume and intensity on the treatment parameters. (**A**) BBBd volume as a function of the applied voltage for 100 pulses. (**B**) BBBd intensity increase as a function of the applied voltage. (**C**). BBBd volume as a function of the number of pulses at 100 and 150 V. (**D**) BBBd intensity increase as a function of the number of pulses at 100 and 150 V.

The dependency of BBBd volume and intensity on the applied voltage was studied in mice treated with 100 pulses at 100, 200 and 300 V (Figure 4). A linear regression was calculated to predict the effect of the applied voltage on the extent of BBBd. A significant regression equation was found (F(1, 19) = 85.60, *p* < 1.8 × 10^−8^) with r^2^ = 0.82. The results suggest that the applied voltage accounted for 82% of the variation in BBBd volume, a large size effect according to Cohen [38]. The prediction equation was BBBd(V) = 0.733 V–22.41, where V is the applied voltage. The results demonstrated a 336.7% increase in the average BBBd volume when the voltage was increased from 0 to 300 V, suggesting strong dependence on the voltage (Figure 5A). The dependency of BBBd intensity (increase in signal intensity over time) on the applied voltage was also studied using liner regression. BBBd intensity increased by 216.2% (from 6.76% ± 0.20% to 14.63% ± 1.75%) when the voltage was increased from 0 to 300 V.

A significant regression equation was also found F(1, 19) = 8.76, *p* < 0.008) with r^2^ = 0.32, suggesting the applied voltage accounted for only 32% of the variation in BBBd intensity. This result is still considered a large effect size according to Cohen, though the results suggest that the relationship between the two variables is weaker than the relationship between the applied voltage and the BBBd volume. The prediction equation was BBBd(V) = 0.022V–8.47, where V is the applied voltage (Figure 5B).

### 3.3. Dependency of BBBd on the Number of Pulses

Linear regressions were calculated to predict the effect of the number of applied pulses on BBBd volume and intensity. Mice were treated using 100, 200, 300 or 400 pulses with applied voltage of either 100 or 150 V. The results demonstrate a 114% and 131% increase in the average volume when the number of pulses was increased from 0 to 300 pulses in the 100 and 150 V treatment groups, respectively. For both 100 and 150 V, significant regression equations were found (F(1, 20) = 15.50, *p* < 0.01) with an r^2^ = 0.44 for 100 V and (F(1, 14) = 24.87, *p* < 0.0002) with an r^2^ = 0.64 for 150 V. The results suggest that the applied voltage accounted for 44% of the variation in BBBd volume in the 100 V groups and 64% in the 150 V groups. Although both effects are considered large, the effect was greater when a higher voltage was applied. The prediction equations were BBBd(P) = 0.15P + 14.09 and BBBd(P) = 0.38P+ 5.24 for 100 and 150 V, respectively, where P is the number of applied pulses. The larger coefficient of the 150 V prediction equation also indicates that increasing the number of pulses at higher voltage has a larger effect (Figure 5C). BBBd intensity increased by 156.20% and 185.06% when the number of pulses was increased from 0 to 400 for 100 and 150 V, respectively. Nevertheless, the linear regression for the 100 V groups was not significant (F(1, 20) = 0.15, *p* < 0.7) with r^2^ = 0.07. This result suggests that although the signal intensity increased significantly between 0 and 100 pulses as described above, increasing the number of pulses did not increase the BBBd intensity. Nevertheless, for the 150 V groups, a significant regression equation was found between the number of applied pulses and BBBd intensity (F(1, 14) = 59.7, *p* < 3 × 10^−4^) with r^2^ = 0.72. These results suggest that the number of pulses accounted for 72% of the variation in BBBd intensity in the 150 V groups (Figure 5D). The results of the regression analysis of BBBd intensity are in accordance with the results of BBBd volume regression analysis, suggesting that when the voltage is increased, the effect of the number of pulses increases as well.

### 3.4. Safety MRI Experiments

**Figure 6:**
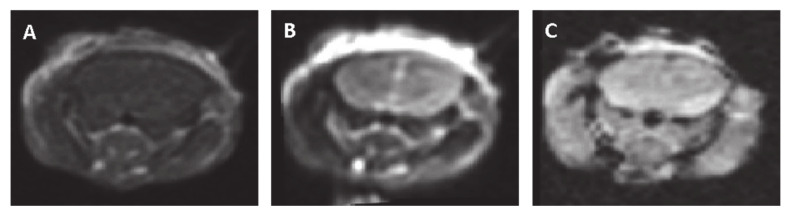
Representative MRI slices obtained 48 h post-pulsed electrical fields (PEFs) application (100 pulses at 300 V) (**A**)**.** Contrast-enhanced T1-MRI. (**B**) T2-MRI. (**C**) Gradient echo (GE) MRI.

In order to evaluate late treatment effects, six mice underwent MRI 48 h post-treatment. No enhancement was observed on the contrast-enhanced T1- MRIs nor hyper-intense regions on the T2-MRIs that might suggest BBBd, edema or tissue damage. No hypo-intense regions were observed on either the GE images or the SWAN images, suggesting no bleedings occurred. Figure 6 shows representative brain slices of a mouse treated with 100 pulses at 300 V (the highest applied voltage).

### 3.5. Evans Blue Extravasation Experiments

**Figure 7:**
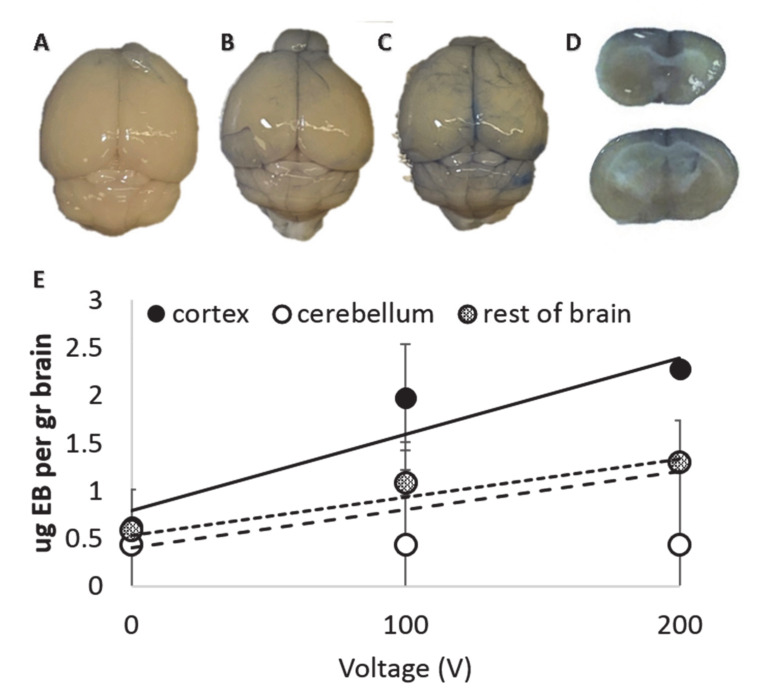
Results of the Evans blue (EB) extravasation experiments. (**A**) A brain of a mouse treated with the sham protocol after 20 min perfusion. (**B**) A brain of a mouse treated with 100 pulses at 100 V. (**C**) A brain of a mouse treated with 100 pulses at 200 V. (**D**) Coronal slices of a mouse treated with 100 (top) and 200 V (bottom). EB is seen mainly in the cortex but can also be seen in the striatum. (**E**) The amount of EB found in the tissue as a function of the applied voltage for the different brain regions.

EB extravasation post-PEFs application was visible to the naked eye compared to the sham brains (Figure 7A–C). Following extraction, the brains were processed for quantification of the extravasation. The results are presented in ¦mg EB per g brain. Similarly to the MRI experiments, three linear regressions were conducted, one for each part of the brain—cortex, cerebellum and the remaining part of the brain. Significant regression equations were found for all three regions, supporting the results of the MRI experiments. The results of the regression are summarized in Table 2 and in Figure 7D. These results clearly demonstrate that the BBB was disrupted as EB does not penetrate the intact BBB.

The results corroborate the results of the MRIs demonstrating stronger BBBd in the cortex than in the rest of the brain. They further demonstrate the treatment ability to enable transport of not only small molecules such as Gd-DOTA, but also macromolecules, as EB binds serum albumin with high affinity [39].

### 3.6. Numerical Model

**Figure 8:**
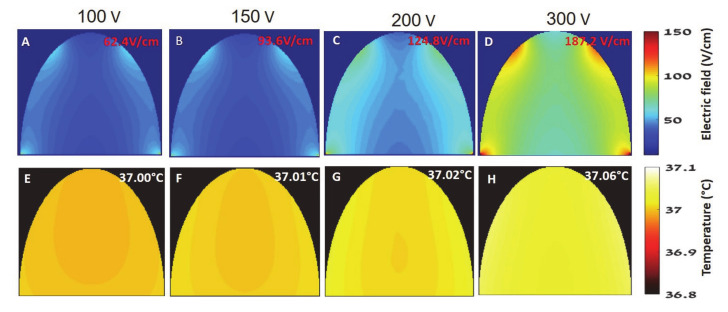
Electric field and temperature distributions after 100 pulses as calculated from the finite elements simulation (**A**–**D**). Electric field distribution (**E**–**H**). Temperature distribution. (**A**) + (**E**) 100 V. (**B**) + (**F**) 150 V. (**C**) + (**G**) 200 V. (**D**) + (**H**) 300 V.

The electric field distribution for the different voltages was calculated using the finite elements model. The results were extracted to Matlab and interpolated to match the MRI resolution. The maximal electric field was extracted for each voltage. The relationship between the applied voltage and maximal electric field in the brain can be described using a linear function:

EF_max_ = 0.624V624V (3)

where EF_max_ is the maximal electric field in the brain and V is the applied voltage. The brain slices with the maximal electric field values are presented in Figure 8A–D. For each applied voltage, the highest electric field values were found in the cortex. This result is in accordance with the MRI and EB experiments demonstrating the stronger treatment effect was found in the cortex. The finite elements model was used to calculate the change in brain temperature since an increase of >1 °C in brain temperature can induce BBBd [40]. There are two mechanisms that may cause a temperature elevation. The first is the Joule heating induced by the electric field in the brain and the second is heat conduction from the less conductive tissues such as the bone and dura. The maximal temperature was determined to be 37.17 °C, i.e., a temperature increase of 0.37 °C. This temperature was achieved after 400 pulses at 150 V. Treatment with 100 pulses at 300 V increased the temperature from 36.8 to only 37.06 °C. The brain slices with the highest temperature for 100 pulses are shown in Figure 8E–H.

## 4. Discussion

The BBB presents a significant obstacle for treating brain diseases such as neurodegenerative disorders and brain tumors [41]. Thus, means to safely disrupt the BBB are of urgent need. We recently demonstrated that short treatments with low PEFs can induce transient BBBd in vitro [23].

Here, our objective was to demonstrate the feasibility of inducing similar BBBd in vivo by non-invasive (intact skull) low PEFs and study the effects of different treatment parameters (applied voltage and number of pulses) on the extent of BBBd. The study was performed in mice. We found that application of 100–400 pulses at 100–300 V can induce subtle BBBd, undetectable by conventional T1-MRI but clearly depicted using the TRAMs. Subtle BBBd using the same treatment protocols with similar dependence on the treatment parameters was also found in the EB experiments.

The TRAMs depicted continuous contrast accumulation over at least 30 min post-contrast injection. The advantage of using delayed-contrast MRI over standard T1-MRI is increased sensitivity to subtle BBBd undetected by conventional MRI techniques [20,24] and the additional information it provides such as time to peak and wash-in/wash-out rates. This additional information may be used for better characterization of the extent of disruption and for determination of the optimal time window for systemic therapeutics administration. Here, we used the maximal intensity increase and the goodness of fit parameters in order to determine BBBd volumes and levels, but in the future, more parameters can be derived from the TRAMs data.

The relationship between the applied voltage and BBBd volume/intensity calculated from the MRIs was studied using linear regressions. The MRI experiments reveled a strong linear relationship between the applied voltage and BBBd volume and a weaker but still significant linear relationship between the applied voltage and BBBd intensity. The EB experiments showed a similar relationship with the amount of dye per gram of brain. Therefore, an important conclusion from the study is that increasing the applied voltage can increase both BBBd volumes and intensities, thus enabling higher therapeutic doses to enter the brain.

Increasing the number of pulses also increased the BBBd volume but to a lesser extent as demonstrated by the smaller coefficient of the prediction equation. Still, the dependence on the number of pulses was found to be stronger when increasing the treatment voltage from 100 to 150 V, suggesting that the relationship is more complex. In the future, a model incorporating both the applied voltage and the number of pulses for simulating low PEFs-induced BBBd should be considered. This can be conducted, for example, by using the Peleg-Fermi model for cell kill probability as a function of treatment voltage and number of pulses, as we have previously conducted when extending the model for EP-induced BBBd [19,42].

The MRI experiments demonstrated the feasibility for passage of small molecules (Gd-DOTA (753.9 Da) across the BBB and that BBBd extent increased with the applied voltage and number of pulses. The results of the EB extravasation experiments not only support the conclusions from the MRI experiments, but also demonstrate that low PEFs induced BBBd, enabling the passage of macromolecules, as EB binds to serum albumin (66 kDa) in the blood.

Both the MRI and the EB extravasation experiments found increased BBBd in the cortex compared to the rest of the brain. These results are in accordance with the finite elements model results showing that the highest electric field was reached in the cortex. Thus, it may be hypothesized that the main parameter affecting the extent of BBBd is the electric field. The maximal electric field in the brain as calculated by the finite elements model for 100 V was 62.4 V/cm and reached 187.2 V/cm for 300 V. It was previously demonstrated by us and others that EP can induce BBBd with electric fields thresholds ranging between 500 and 700 V/cm [17,19]. Recently, Lorenzo et al. [21] demonstrated that H-FRE also induces BBBd, at a lower threshold (113 V/cm), when using two needle electrodes inserted into the brain parenchyma with a voltage to distance ratio of 600 V/cm. Although determining the electric field threshold for BBBd was beyond the scope of this study, the maximal electric fields induced in the brain using our closed cranium setup were significantly smaller (62.4 V/cm for 100 V) by an order of magnitude than those previously described in the literature as the threshold for EP and by a factor of 2 than the H-FRE threshold.

One explanation for these differences can be the increased sensitivity of the TRAMs to subtle BBBd compared to conventional MRI. Another explanation may be that the mechanism for inducing BBBd using low PEFs is different than EP. In addition, those EP and H-FRE experiments were conducted using either one or two needle electrodes inserted into the brain through burr holes drilled in the skull. This method of inducing BBBd requires induction of high electric fields in the brain which may cause, in addition to BBBd, tissue damage in smaller volumes. Even if no irreversible EP occurs, damage along the electrodes path is expected [21].

On top of that, exposure to high electric fields can induce significant vasodilatation which can, by itself, induce BBBd but also increase wash-out rates of therapeutic agents [20], thus diminishing the efficacy of the disruption. We previously applied TRAMs for studying the effects of point source EP (EP induced by a minimally invasive setup consisting of one partially insulated intracranial needle and one external surface electrode) [12,17,18,21]. In the point source study, EP-induced BBBd appeared as an enhancing region on standard T1-MRI and was depicted as blue in the TRAMs (negative signal), consistent with vasodilatation and fast contrast clearance rates. The enhancing region was surrounded by a thin red rim in the TRAMs, representing contrast accumulation.

In the current study, the subtle BBBd induced by low PEFs did not show any enhancement on standard T1-MRI and was depicted red in the TRAMs. This suggests that EP-induced BBBd is depicted differently on TRAMs than low PEFs-induced subtle BBBd. Although the mechanism of action of low PEFs-induced BBBd remains unclear, in vitro studies demonstrated that EP is not part of the mechanism of action. It has been suggested that low PEFs induce BBBd via the paracellular pathway, by affecting the tight junctions and adherent junctions proteins [23,43], although the exact mechanism of action remains unclear.

The results of the MRIs acquired 48 h post-treatment reveal no signs of hemorrhage, edema or damage. Nevertheless, these results do not rule out microscale damage to the tissue or microcirculation which may be below the sensitivity of the MRI. Thus, a comprehensive histological study, using brains extracted at different time points post-treatment, is needed to evaluate both early and late possible tissue changes and damage.

It is important to note that we address our results as demonstrating the feasibility for inducing BBBd non-invasively since the treatment was performed extracranially, i.e., without opening the skull, but was not non-invasive. The skin of the mice was cut open to allow the electrodes to be in direct contact with the intact skull. This was conducted in order to reduce the loss in the electric field strength in the non-conducting skin and fur due to technical limitations of our experimental system. Still, it is possible to achieve similar electric fields in the brain without opening the skin by, for example, increasing the applied voltage or changing the electrode setup. Further research and development are needed to achieve a completely non-invasive treatment protocol suitable for clinical application.

Non-invasiveness is a major advantage of low PEFs-induced BBBd. An additional advantage is the short treatment duration (only several minutes). In the future, these advantages may enable the development of safe treatment protocols combined with systemic drug administration for CNS disorders that can be repeated as needed.

## 5. Conclusions

Our results demonstrate the feasibility of applying low PEFs non-invasively for inducing subtle yet significant BBBd. A significant linear relationship was found between the extent of BBBd and the applied voltage and number of pulses. EB extravasation was also found in the brain post-low PEFs application, suggesting that disruption was not limited to small molecules. Our results suggest significantly lower electric field thresholds for BBBd than previously reported and they present the first proof of concept for a non-invasive application of PEFs-induced BBBd. As the treatment is rapid and may be applied in a non-invasive manner, it may be used repeatedly in parallel to systemic drug administration for efficient delivery of therapeutic agents into the brain. In the future, the efficacy of combining non-invasive low PEFs with drug therapy for CNS disorders such as neurodegenerative diseases and brain tumors should be evaluated.

## 6. Patents

Pending PCT #WO2019175871A1 titled “method for changing blood brain barrier permeability” was filed based on the study results.

## Figures and Tables

**Table 1:** Tissue electric and thermal properties.

	Brain	Dura	Skull
Thermal conductivity W/(m*K)	0.565 [31]	0.44 [32]	0.16 [31]
Heat capacity J/(kg*K)	3650 [33]	3364 [32]	1700 [31]
Density Kg/m^3^	1040 [31]	1174 [32]	1500 [31]
Blood perfusion rate mL/(s*cm^3^)	0.007 [33]	0.143 × 10^−3^ [31]	0.143 × 10^−3^ [31]
Metabolic heat production W/m^3^	10437 [33]	4144 [32]	70 [31]
Electric conductivity s/m	0.258 [34]	0.06 [32]	0.01 [35]

**Table 2:** Results of linear regression analysis for the three brain regions.

Brain Region	r^2^	Significance	Prediction Equation	Fold Increase 0–100	Fold Increase 0–200
Cortex	0.54	0.006	BBBd(V) = 0.008 V + 0.8	3.18	3.66
Cerebellum	0.81	0.0002	BBBd(V) = 0.004 + 0.40	1.63	3.05
Rest of brain	0.67	0.001	BBBd(V) = 0.004 + 0.53	1.85	2.22

## Data Availability

The data presented in this study including full MRI scans are available on request from the corresponding author.
